# Synthesis and antimalarial potential of some novel quinoline-pyrazolopyridine derivatives

**DOI:** 10.17179/excli2016-677

**Published:** 2016-11-28

**Authors:** Deepika Saini, Sandeep Jain, Ajay Kumar, Neelam Jain

**Affiliations:** 1 Drug Discovery and Research Laboratory, Department of Pharmaceutical Sciences, Guru Jambheshwar University of Science and Technology, Hisar-125001; 2 Institute of Pharmaceutical Sciences, Kurukshetra University, Kurukshetra 136119; 3 Department of Pharmaceutical Education and Research, Bhagat Singh Phool Mahila Vishwavidyalaya, Khanpur Kalan, Sonepat-131305

**Keywords:** quinoline, pyrazolopyridine, cyanopyridine, antimalarial activity, Plasmodium

## Abstract

A series of 1-(4-methylquinolin-2-yl)-4,6-diaryl-1H-pyrazolo[3,4-b]pyridin-3-amine derivatives was synthesized by the reaction of 3-cinnamoyl-4-hydroxy-6-methyl-2*H*-pyran-2-ones with 2-chloro-4,6-diphenylnicotinonitrile analogues in the presence of 2-hydrazino-4-methyl quinoline and ethanol. The newly synthesized compounds were characterized by IR, ^1^H NMR and mass spectral data. The synthetic series of novel quinoline-pyrazolopyridine hybrids were screened for *in vitro *schizont maturation assay against chloroquine sensitive 3D7 strain of *Plasmodium falciparum*, from which the most five active analogues were further evaluated for *in vivo *4-day suppressive test in Swiss albino mice. Among the series, **5p** (containing 4-Cl substituent attached to both aryl ring) portrayed considerable potent antimalarial activity during *in vitro *as well as *in vivo *study.

## Introduction

Malaria is a mosquito-borne disease caused by Plasmodium parasites that infects and destroys red blood cells, leading to fever, severe anaemia, cerebral malaria and if untreated, it even results in death (Baragana et al., 2011[1]). Malaria remains one of the critical global health problems, with 214 million cases causing 438,000 deaths in 2015, according to WHO estimates. Particularly susceptible groups include pregnant women and young children (Boudhar et al., 2016[2]). Chemotherapeutic cure of malaria is becoming progressively more challenging with the fast development of resistance of Plasmodium falciparum, the most vicious and lethal malaria parasite, to many of the standard amine antimalarial drugs like chloroquine and artesunate (Miura et al.,2010[11]; Gupta et al., 2010[5]; Dondrop et al., 2009[3]). Therefore, an urgent need exists for the development of new simple, safe, and more effective antimalarial drugs.

Quinoline nucleus has been extensively employed as core scaffold in the evolution of anti-malarial agents. Substituted quinolones derivatives are considered as remarkable tools for the eradication of malaria. In order to overcome the challenge of resistance, various heterocycles have also been explored to develop new anti-malarial regents, pyrazolopyridines being one of them (Kumar et al., 2014[9]). Hybridization concept has been proved as a lead for developing new anti-malarial active agents (Menezes et al., 2002[10]; Silva et al., 2016[14]). In the present study, we have developed hybrids of pyrazolopyridine based quinoline for anti-malarial potential. 

## Materials and Methods

### Chemistry

**Figure 1:**
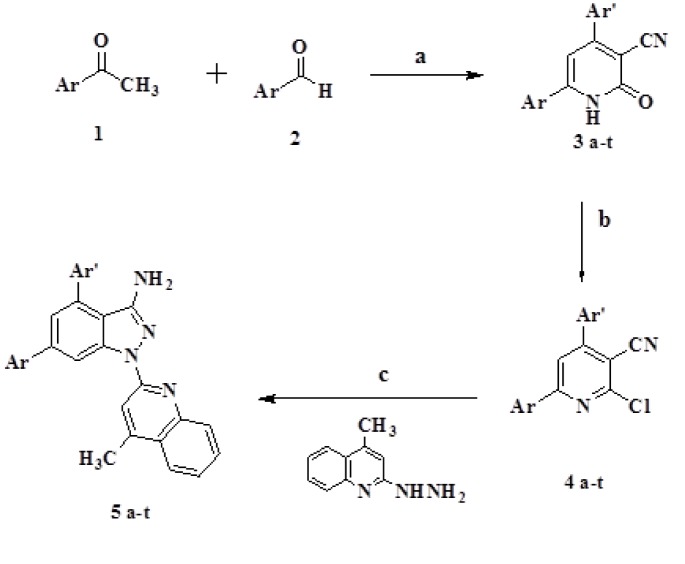
Synthesis of quinoline-pyrazolopyridine analogues 5a-t. Reagents and Conditions: a) Ethyl cyanoacetate, Ammonium acetate, BuOH, reflux, 3 hr. b) POCl_3_, PCl_5_, Δ, 3 hr. c) EtOH, reflux, 12 hr

The synthetic procedure is given in Figure 1(Fig. 1).

#### Synthesis of 2-oxo-4, 6-diphenyl-1,2-dihydropyridine-3-carbonitrile derivatives (3 a-t)

Synthesis was carried out as per procedure reported in literature (Jain et al., 1995[8]). A mixture of aromatic aldehydes, ketones, ethyl cyanoacetate (0.01 mol) and ammonium acetate (0.08 mol) in n-butanol (20 ml) was refluxed for 3 h. The obtained precipitate was filtered and washed successively with ethanol.

#### Synthesis of 2-chloro-4,6-diphenylnicotinonitrile derivatives (4 a-t)

A suspension of pyridone compound 1, phosphorus pentachloride and phosphorus oxychloride was heated on a water bath for 3 h. The reaction mixture was poured gradually into ice-cold water and neutralized by dilute ammonia solution. The separated solid was filtered and recrystallized from ethanol to afford the corresponding chloro derivative (Hamdy and Gamal-Eldeen, 2009[7]).

#### Synthesis of 1-(4-methylquinolin-2-yl)-4,6-diaryl-1H-pyrazolo[3,4-b]pyridin-3-amine derivatives (5 a-t)

The target compounds were synthesized by reported procedure (Hamdy and Gamal-Eldeen, 2013[6]). Chloro derivatives 4(a-t) (10 mmol) in absolute ethanol (30 ml) was added with excess of 2-hydrazino-4-methyl quinoline (10 mmol) and the mixture was refluxed for 12 h. The solution was cooled, filtered and recrystallized from acetic acid afforded 5(a-t).

#### 1-(4-methylquinolin-2-yl)-4,6-diphenyl-1H-pyrazolo[3,4-b]pyridin-3-amine (5a)

Yield- 66 %; M.pt.- 150 °C; IR- NH_2_ str (3451, 3343), =C-H Str (3050), C=N (1600); ^1^HNMR (CDCl_3_) δ- 8.19 (s, 1H, 3^rd^ position of quinoline), 7.1-7.8 (m, 15H, aromatic ring), 5.6 (s, 2H, NH_2_), 2.61 (s, 3H, CH_3_ of quinoline); m/e- 428.34 (M+1); Calculated- C=78.67, H=4.95, N=16.38; found C=78.59, H=4.32, N=16.42.

#### 6-(4-fluorophenyl)-1-(4-methylquinolin-2-yl)-4-phenyl-1H-pyrazolo[3,4-b]pyridin-3-amine (5b)

Yield- 69 %; M.pt.- 156-158 °C; IR- NH_2_ str (3450, 3347), =C-H Str (3053), C=N (1602), C-F (600);^ 1^HNMR (CDCl_3_) δ- 8.32 (s, 1H, 3^rd^ position of quinoline), 7.2-7.7 (m, 14H, aromatic ring), 5.4 (s, 2H, NH_2_), 2.63 (s, 3H, CH_3_ of quinoline); m/e- 446.23 (M+1); Calculated- C=75.49, H=4.53, N=15.72; found C=75.23, H=4.46, N=15.65.

#### 6-(4-methoxyphenyl)-1-(4-methylquinolin-2-yl)-4-phenyl-1H-pyrazolo[3,4-b]pyridin-3-amine (5c)

Yield- 72 %; M.pt.- 163-166 °C; IR- NH_2_ str (3455, 3346), =C-H Str (3048), C=N (1605), C-O-C (1152); ^1^HNMR (CDCl_3_) δ- 8.24 (s, 1H, 3^rd^ position of quinoline), 7.3-7.9 (m, 14H, aromatic ring), 5.6 (s, 2H, NH_2_), 3.82 (s, 3H, OCH_3_), 2.65 (s, 3H, CH_3_ of quinoline); m/e-458.23 (M+1); Calculated- C=76.13, H=5.07, N=15.31; found C=76.46, H=5.03, N=15.67.

#### 1-(4-methylquinolin-2-yl)-4-phenyl-6-(thiophen-2-yl)-1H-pyrazolo[3,4-b]pyridin-3-amine (5d)

Yield- 65 %; M.pt.- 165-167 °C; IR- NH_2_ str (3460, 3350), =C-H Str (3056), C=N (1604); ^1^HNMR (CDCl_3_) δ- 8.23 (s, 1H, 3^rd^ position of quinoline), 7.2-7.7 (m, 13H, aromatic ring), 5.4 (s, 2H, NH_2_), 2.66 (s, 3H, CH_3_ of quinoline); m/e- 434.45 (M+1); Calculated- C=72.03. H=4.42, N=16.15; found C=72.12, H=4.56, N=16.23.

#### 1-(4-methylquinolin-2-yl)-6-phenyl-4-p-tolyl-1H-pyrazolo[3,4-b]pyridin-3-amine (5e)

Yield- 69 %; M.pt.- 158-160 °C; IR- NH_2_ str (3453, 3339), =C-H Str (3054), C=N (1603); ^1^HNMR (CDCl_3_) δ- 8.15 (s, 1H, 3^rd^ position of quinoline), 7.3-7.9 (m, 14H, aromatic ring), 5.4 (s, 2H, NH_2_), 2.59 (s, 3H, CH_3_ of quinoline), 2.23 (s, 3H, CH_3_); m/e- 442.64 (M+1); Calculated- C=78.89, H=5.25, N=15.86; found C=78.83, H=5.27, N=15.84.

#### 6-(4-fluorophenyl)-1-(4-methylquinolin-2-yl)-4-p-tolyl-1H-pyrazolo[3,4-b]pyridin-3-amine (5f)

Yield- 68 %; M.pt.- 160-162 °C; IR- NH_2_ str (3462, 3346), =C-H Str (3053), C=N (1602), C-F (602); ^1^HNMR (CDCl_3_) δ- 8.23 (s, 1H, 3^rd^ position of quinoline), 7.2-7.7 (m, 13H, aromatic ring), 5.4 (s, 2H, NH_2_), 2.66 (s, 3H, CH_3_ of quinoline), 2.13 (s, 3H, CH_3_); m/e- 460.34 (M+1); Calculated- C=75.80, H=4.83, N=15.24; Found- C=75.84, H=4.86, N=15.30.

#### 6-(4-methoxyphenyl)-1-(4-methylquinolin-2-yl)-4-p-tolyl-1H-pyrazolo[3,4-b]pyridin-3-amine (5g)

Yield- 70 %; M.pt.- 163-165 °C; IR- NH_2_ str (3459, 3343), =C-H Str (3057), C=N (1603), C-O-C (1162); ^1^HNMR (CDCl_3_) δ- 8.18 (s, 1H, 3^rd^ position of quinoline), 7.1-7.9 (m, 13H, aromatic ring), 5.7 (s, 2H, NH_2_), 3.86 (s, 3H, OCH_3_), 2.58 (s, 3H, CH_3_ of quinoline), 2.27 (s, 3H, CH_3_); m/e- 472.23 (M+1); Calculated- C=76.41. H=5.34, N=14.85; Found- C=76.25, H=5.47, N=14.88.

#### 1-(4-methylquinolin-2-yl)-6-(thiophen-2-yl)-4-p-tolyl-1H-pyrazolo[3,4-b]pyridin-3-amine (5h) 

Yield- 70 %; M.pt.- 155-157 °C; IR- NH_2_ str (3450, 3347), =C-H Str (3052), C=N (1607); ^1^HNMR (CDCl_3_) δ- 8.24 (s, 1H, 3^rd^ position of quinoline), 7.2-7.9 (m, 12H, aromatic ring), 5.7 (s, 2H, NH_2_), 2.52 (s, 3H, CH_3_ of quinoline), 2.26 (s, 3H, CH_3_); m/e-448.45 (M+1); Calculated- C=72.46, H=4.73, N=15.65; Found- C=72.48, H=5.42, N=15.63.

#### 1-(4-methylquinolin-2-yl)-4,6-dip-tolyl-1H-pyrazolo[3,4-b]pyridin-3-amine (5i)

Yield- 70 %; M.pt.- 161-163 °C; IR- NH_2_ str (3451, 3343), =C-H Str (3050), C=N (1600); ^1^HNMR (CDCl_3_) δ- 8.23 (s, 1H, 3^rd^ position of quinoline), 7.1-7.8 (m, 13H, aromatic ring), 5.2 (s, 2H, NH_2_), 2.52 (s, 3H, CH_3_ of quinoline), 2.21 (s, 3H, CH_3_), 2.26 (s,3H, CH_3_); m/e-456.34 (M+1); Calculated- C=79.10, H=5.53, N=15.37; Found- C=79.15, H=5.58, N=15.40.

#### 4-(4-methoxyphenyl)-1-(4-methylquinolin-2-yl)-6-phenyl-1H-pyrazolo[3,4-b]pyridin-3-amine (5j)

Yield- 68 %; M.pt.- 148-150 °C; IR- NH_2_ str (3450, 3342), =C-H Str (3045), C=N (1602), C-O-C (1155); ^1^HNMR (CDCl_3_) δ- 8.16 (s, 1H, 3^rd^ position of quinoline), 7.1-7.7 (m, 14H, aromatic ring), 5.3 (s, 2H, NH_2_), 3.81 (s, 3H, OCH_3_), 2.62 (s, 3H, CH_3_ of quinoline); m/e-458.46 (M+1); Calculated- C=76.13, H=5.07, N=15.31; found C=76.36, H=5.08, N=15.60.

#### 6-(4-fluorophenyl)-4-(4-methoxyphenyl)-1-(4-methylquinolin-2-yl)-1H-pyrazolo[3,4-b]pyridin-3-amine (5k)

Yield- 70 %; M.pt.- 167-169 °C; IR- NH_2_ str (3453, 3350), =C-H Str (3043), C=N (1610), C-O-C (1170), C-F (600); ^1^HNMR (CDCl_3_) δ- 8.16 (s, 1H, 3^rd^ position of quinoline), 7.1-7.7 (m, 13H, aromatic ring), 5.3 (s, 2H, NH_2_), 3.81 (s, 3H, OCH_3_), 2.62 (s, 3H, CH_3_ of quinoline); m/e-476.64 (M+1); Calculated- C=73.25, H=4.66, N=14.73; Found- 73.56, H=4.65, N=14.86.

#### 4,6-bis(4-methoxyphenyl)-1-(4-methylquinolin-2-yl)-1H-pyrazolo[3,4-b]pyridin-3-amine (5l)

Yield- 70 %; M.pt.- 170-172 °C; IR- NH_2_ str (3455, 3346), =C-H Str (3055), C=N (1605), C-O-C (1165); ^1^HNMR (CDCl_3_) δ- 8.25 (s, 1H, 3^rd^ position of quinoline), 7.2-7.8 (m, 13H, aromatic ring), 5.8 (s, 2H, NH_2_), 3.83 (s, 3H, OCH_3_), 3.87 (s, 3H, OCH_3_), 2.54 (s, 3H, CH_3_ of quinoline); m/e- 488.53 (M+1); Calculated- C=73.90. H=5.17, N=14.36; Found- C=73.85, H=5.37, N=14.24.

#### 4-(4-methoxyphenyl)-1-(4-methylquinolin-2-yl)-6-(thiophen-2-yl)-1H-pyrazolo[3,4-b]pyridin-3-amine (5m)

Yield- 62 %; M.pt.- 169-171 °C; IR- NH_2_ str (3451, 3344), =C-H Str (3051), C=N (1600); ^1^HNMR (CDCl_3_) δ- 8.28 (s, 1H, 3^rd^ position of quinoline), 7.1-7.7 (m, 12H, aromatic ring), 5.6 (s, 2H, NH_2_), 3.83 (s, 3H, OCH_3_), 2.51 (s, 3H, CH_3_ of quinoline); m/e- 464.18 (M+1); Calculated- C=69.96, H=4.57, N=15.11; Found- C=69.86, H=4.64, N=15.18.

#### 4-(4-methoxyphenyl)-1-(4-methylquinolin-2-yl)-6-p-tolyl-1H-pyrazolo[3,4-b]pyridin-3-amine (5n)

Yield- 65 %; M.pt.- 160-162 °C; IR- NH_2_ str (3446, 3325), =C-H Str (3055), C=N (1600), C-O-C (1165); ^1^HNMR (CDCl_3_) δ- 8.46 (s, 1H, 3^rd^ position of quinoline), 7.2-7.8 (m, 13H, aromatic ring), 5.5 (s, 2H, NH_2_), 3.80 (s, 3H, OCH_3_), 2.53 (s, 3H, CH_3_ of quinoline), 2.19 (s, 3H, CH_3_); m/e- 472.54 (M+1); Calculated- C=76.41. H=5.34, N=14.85; Found- C=76.44, H=5.40, N=14.86.

#### 4-(4-chlorophenyl)-1-(4-methylquinolin-2-yl)-6-phenyl-1H-pyrazolo[3,4-b]pyridin-3-amine (5o)

Yield- 68 %; M.pt.- 152-154 °C; IR- NH_2_ str (3450, 3348), =C-H Str (3055), C=N (1604), C-Cl (658); ^1^HNMR (CDCl_3_) δ- 8.13 (s, 1H, 3^rd^ position of quinoline), 7.2-7.5 (m, 14H, aromatic ring), 5.9 (s, 2H, NH_2_), 2.62 (s, 3H, CH_3_ of quinoline); m/e- 462.96 (M+1); Calculated- C=72.80, H=4.36, N=15.16; found C=72.76, H=4.28, N=15.10.

#### 4-(4-chlorophenyl)-6-(4-fluorophenyl)-1-(4-methylquinolin-2-yl)-1H-pyrazolo[3,4-b]pyridin-3-amine (5p)

Yield- 67 %; M.pt.- 160-163 °C; IR- NH_2_ str (3451, 3350), =C-H Str (3051), C=N (1603), C-Cl (656), C-F (602); ^1^HNMR (CDCl_3_) δ- 8.26 (s, 1H, 3^rd^ position of quinoline), 7.3-7.9 (m, 13H, aromatic ring), 5.2 (s, 2H, NH_2_), 2.59 (s, 3H, CH_3_ of quinoline); m/e- 480.96 (M+1); Calculated- C=70.07, H=3.99, N=14.59; found C=70.12, H=3.95, N=14.54.

#### 4-(4-chlorophenyl)-6-(4-methoxyphenyl)-1-(4-methylquinolin-2-yl)-1H-pyrazolo[3,4-b]pyridin-3-amine (5q)

Yield- 67 %; M.pt.- 160-163 °C; IR- NH_2_ str (3451, 3350), =C-H Str (3051), C=N (1603), C-Cl (660);

^1^HNMR (CDCl_3_) δ- 8.15 (s, 1H, 3^rd^ position of quinoline), 7.2-7.9 (m, 13H, aromatic ring), 5.1 (s, 2H, NH_2_), 3.82 (s, 3H, OCH_3_), 2.54 (s, 3H, CH_3_ of quinoline); m/e- 492.56 (M+1); Calculated- C=70.80, H=4.51, N=14.24; found C=70.69, H=3.94, N=14.54.

#### 4-(4-chlorophenyl)-1-(4-methylquinolin-2-yl)-6-p-tolyl-1H-pyrazolo[3,4-b]pyridin-3-amine (5r)

Yield- 69 %; M.pt.- 164-166 °C; IR- NH_2_ str (3458, 3354), =C-H Str (3053), C=N (1608), C-Cl (661);

^1^HNMR (CDCl_3_) δ- 8.23 (s, 1H, 3^rd^ position of quinoline), 7.3-7.9 (m, 13H, aromatic ring), 5.5 (s, 2H, NH_2_), 2.54 (s, 3H, CH_3_ of quinoline, 2.14 (s, 3H, CH_3_); m/e- 476.44 (M+1); Calculated- C=73.18. H=4.66, N=14.71; Found- C=73.42, H=4.68, N=14.80.

#### 4-(4-fluorophenyl)-1-(4-methylquinolin-2-yl)-6-phenyl-1H-pyrazolo[3,4-b]pyridin-3-amine (5s)

Yield- 64 %; M.pt.- 156-159 °C; IR- NH_2_ str (3451, 3346), =C-H Str (3051), C=N (1600), C-F (604); ^1^HNMR (CDCl_3_) δ- 8.20 (s, 1H, 3^rd^ position of quinoline), 7.1-7.7 (m, 14H, aromatic ring), 5.2 (s, 2H, NH_2_), 2.61 (s, 3H, CH_3_ of quinoline); m/e- 446.46 (M+1); Calculated- C=75.49, H=4.54, N=15.72; found C=75.46, H=4.51, N=15.70.

#### 4-(4-fluorophenyl)-1-(4-methylquinolin-2-yl)-6-p-tolyl-1H-pyrazolo[3,4-b]pyridin-3-amine (5t)

Yield- 66 %; M.pt.- 162-164 °C; IR- NH_2_ str (3461, 3338), =C-H Str (3055), C=N (1601), C-F (604); ^1^HNMR (CDCl_3_) δ- 8.21 (s, 1H, 3^rd^ position of quinoline), 7.1-7.7 (m, 13H, aromatic ring), 5.6 (s, 2H, NH_2_), 2.61 (s, 3H, CH_3_ of quinoline), 2.12 (s, 3H, CH_3_); m/e- 460.54 (M+1);Calculated- C=75.80, H=4.83, N=15.24; Found- C=75.82, H=4.81, N=15.28.

### In vitro antimalarial activity

The schizont maturation assay was used to evaluate the *in vitro* activity of synthesized series (Pandey et al., 2016[12]). In schizontocidal assay of test samples, parasitized blood was made by infecting red blood cells with CQ-S (3D7) strain of *P. falciparum* with 2-3 % parasitemia in RPMI 1640 medium. The parasite cultures, prior to experimentation, were synchronized by treatment with 5 % D-sorbitol. Stock solutions of samples were prepared separately for all the synthetic derivatives, diluting with DMSO to a concentration of 1 mg/ml. Serial double dilutions of each set of test compounds were made in 96 well microtiter plates with concentration ranging from 1.8-500 μg/ml against a control containing the incomplete medium with same concentration of DMSO. In each well 100 μl of the diluted test compound, 10 μl parasitized blood (4 - 5 % rings) in 100 μl incomplete medium. Four wells of the last row were set as general controls to check the normal growth of the parasite. The plates were incubated at 37 °C in gas mixture of 90 % N_2_, 5 % CO_2_ and 5 % O_2_ for 24 h. A thick blood smear was prepared from all wells and stained with giemsa stain. Numbers of schizonts were counted per 200 asexual stage parasites. The values were compared between test and control wells.

### In vivo antimalarial activity

Acute toxicity testing was performed as per OECD-423 (Ecobichon, 1977[4]). The animals were checked for any abnormal behavior for 30 minutes and then after every 4 h. After calculating LD_50_, the *in vivo* antimalarial efficacy was determined by Peter's 4 day suppressive test (Peters et al., 1975[13]). Malaria infection was established in mice by inoculatation on the first day (day 0), intraperitoneally, with 0.2 ml of infected blood containing about 1×10^7 ^*P. berghei *parasitized erythrocytes. The animals were divided into groups of five mice each and orally administered, shortly after inoculation, with 200 mg/kg doses of test compounds for 4 consecutive days. Chloroquine (5mg/kg body weight) was administered positive (standard) control group and negative control group were administered the same amount of solvent used to suspend the test compound. On the fourth day, blood was withdrawn from tail vein of mice. Thin smears were made, fixed with methanol and stained with Giemsa. Parasitemia was examined microscopically and percent suppression was subsequently calculated. 

## Results and Discussion

### Chemistry

Substituted acetophenones underwent cyano condensation reaction with different aromatic aldehyde, ethyl cyanoacetate and excess of ammonium acetate in n-butanol to give corresponding 3(a-t). IR spectrum of compounds confirmed the synthetic product by exhibiting absorption band at 3439 due to NH group, 2223 due to CN and 1640 due to C=O group. ^1^H NMR also showed singlet signal at 12.1 due to NH protons. Further heating compounds 3(a-t) with phosphorous oxychloride and phosphorous pentachloride led to the synthesis of 2-chloro pyridines 4(a-t) which upon further reaction with 2-hydrazino-4-methyl-quinoline afforded pyrazolo pyridine derivatives 5(a-t). The structure of target compounds were confirmed by their elemental analysis and spectral data.IR spectrum displayed band at 3454 and 3342 for NH_2_ and band for C=N at 1601 cm^-1^. One more characteristic band C=N near 2200 cm^-1^ disappeared.

### Biological activity

#### In vitro antimalarial activity

All the synthesized 20 compounds were screened *in vitro* for their anti-malarial potential using schizont maturation inhibition. EC_50_ values for all the compounds are enlisted in Table 1(Tab. 1). The activities of compounds appeared in the following order:

5p > 5k > 5q > 5b > 5s > 5o > 5l > 5j > 5c > 5a > 5m > 5t > 5r > 5f > 5d > 5n > 5g > 5e > 5h > 5i.

The results revealed that the compounds with halogen were having moderate to good activity. Among the series, compound 5p, which was substituted with chalcone and fluorine at para position of two phenyl rings, was found to be the best. Similarly compounds 5b, 5k, 5o, 5q and 5s displayed good anti-malarial activity with EC_50 _1.921-2.916 µg/ml. All these compounds were substituted with halogen on at least one phenyl ring. Results also revealed that the presence of -CH_3 _group led to the reduction in anti-malarial potential. Compound 5i was found to be the least effective with EC_50 _10.929 µg/ml. Compounds 5b, 5k, 5p, 5q, and 5s were further screened for their *in vivo* anti-malarial potential.

#### In vivo antimalarial activity

Dose was calculated by acute toxicity studies. All the compounds were found to possess dosage of 200 mg/kg. Further compounds were tested *in vivo* for anti-malarial activity by 4 day suppression. Result for each compound has been displayed in table 2(Tab. 2). Compound 5p was found to be the most effective with %age suppression of 60.25 % while standard treatment cured all the five animals with 100 % suppression and 100 % survival rate. On 7^th^ day, 4 out of 5 mice remained alive using treatment with the test compound 5p. The order of activity was observed as 5p > 5k > 5q > 5b > 5s.

The results divulged that presence of halogens on both the rings led to highest suppression of parasitemia.

## Conclusion

In the present research work, we have synthesized different derivatives of cyanopyridines which were effectively converted into quinoline based pyrazolopyridine derivatives. Most of the compounds displayed significant potential for anti-malarial activity. Compound 5p exhibited best *in vitro* as well as *in vivo* anti-malarial activity among the series and was comparable with the standard chloroquine. These results indicate that the pyrazolopyridine-quinoline hybrids may be promising leads and may also be proved as significant model for further structural as well as biological optimization.

## Conflict of interest

The authors report no conflict of interest.

## Acknowledgement

The authors are thankful to Department of Science and Technology, New Delhi for providing financial assistance.

## Figures and Tables

**Table 1:**
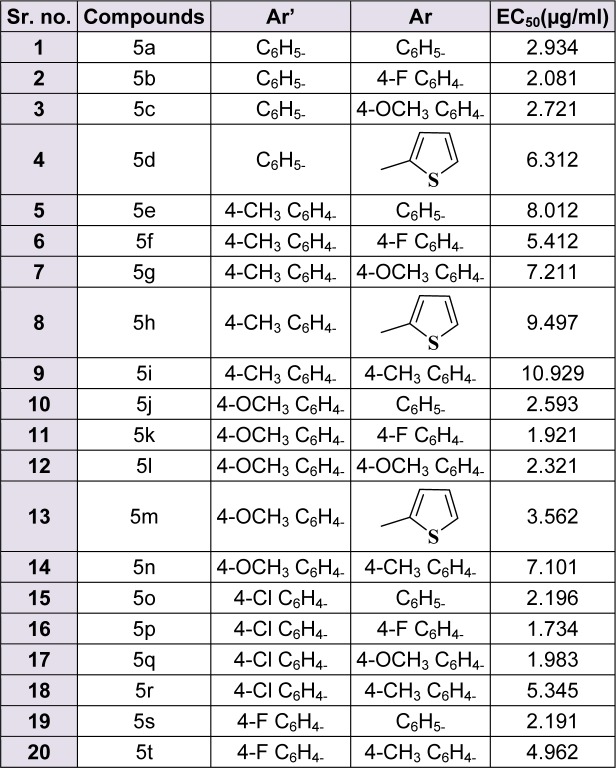
*In Vitro *antimalarial activity of synthetic derivatives 5(a-t) against CQ-Sensitive 3d7 strain of *P. falciparum*

**Table 2:**
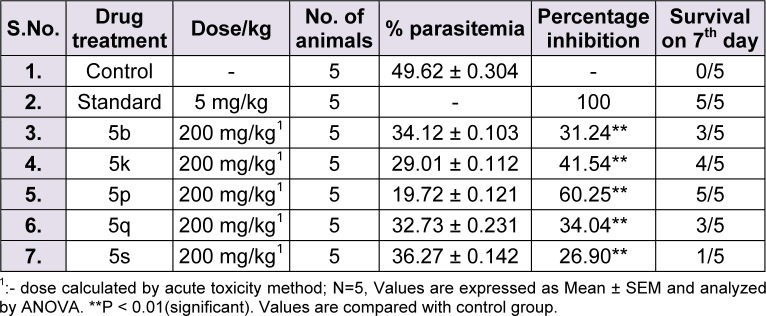
*In vivo *antimalarial activity of 5 selected compounds against *P. berghei *strain in Swiss albino mice
